# Transgenic *Kalanchoë blossfeldiana*, Containing Individual *rol* Genes and Open Reading Frames Under *35S* Promoter, Exhibit Compact Habit, Reduced Plant Growth, and Altered Ethylene Tolerance in Flowers

**DOI:** 10.3389/fpls.2021.672023

**Published:** 2021-05-07

**Authors:** Bruno Trevenzoli Favero, Yi Tan, Yan Lin, Hanne Bøge Hansen, Nasim Shadmani, Jiaming Xu, Junou He, Renate Müller, Aldo Almeida, Henrik Lütken

**Affiliations:** ^1^Section for Crop Sciences, Department of Plant and Environmental Sciences, Faculty of Science, University of Copenhagen, Taastrup, Denmark; ^2^Section for Plant Biochemistry, Department of Plant and Environmental Sciences, Faculty of Science, University of Copenhagen, Frederiksberg, Denmark

**Keywords:** *Agrobacterium rhizogenes*, cellular localization, compact plant, flower longevity, postharvest performance, root oncogenic loci, ΔORF13a, ORF14

## Abstract

Reduced growth habit is a desirable trait for ornamental potted plants and can successfully be obtained through *Rhizobium rhizogenes* transformation in a stable and heritable manner. Additionally, it can also be obtained by transformation with *Agrobacterium tumefaciens* harboring specific genes from *R. rhizogenes*. The bacterial T-DNA harbors four *root oncogenic loci* (*rol*) genes and 14 less known open reading frames (ORFs). The four *rol* genes, i.e., *rol*A, *rol*B, *rol*C, and *rol*D, are conceived as the common denominator for the compact phenotype and the other less characterized ORFs seem auxiliary but present a potential breeding target for less aberrant and/or more tailored phenotypes. In this study, *Kalanchoë blossfeldiana* ‘Molly’ was transformed with individual *rol* genes and selected ORFs in *35S* overexpressing cassettes to comprehensively characterize growth traits, gene copy and expression, and ethylene tolerance of the flowers. An association of reduced growth habit, e.g. height and diameter, was observed for *rol*B2 and ORF14-2 when a transgene single copy and high gene expression were detected. Chlorophyll content was reduced in overexpressing lines compared to wild type (WT), except for one ΔORF13a (a truncated ORF13a, where SPXX DNA-binding motif is absent). The flower number severely decreased in the overexpressing lines compared to WT. The anthesis timing showed that WT opened the first flower at 68.9 ± 0.9 days and the overexpressing lines showed similar or up to 24 days delay in flowering. In general, a single or low relative gene copy insertion was correlated to higher gene expression, ca. 3 to 5-fold, in *rol*B and ΔORF13a lines, while in ORF14 such relation was not directly linked. The increased gene expression observed in *rol*B2 and ΔORF13a-2 contributed to reducing plant growth and a more compact habit. Tolerance of detached flowers to 0.5 μl L^−1^ ethylene was markedly higher for ORF14 with 66% less flower closure at day 3 compared to WT. The subcellular localization of *rol*C and ΔORF13a was investigated by transient expression in *Nicotiana benthamiana* and confocal images showed that *rol*C and ΔORF13a are soluble and localize in the cytoplasm being able to enter the nucleus.

## Introduction

*Rhizobium rhizogenes*-mediated plant transformation has for the last 4 decades been intensively applied in many studies with the pursuit of introducing compact habit and reduced plant growth traits in plants, primarily ornamentals (Christensen et al., 2008; Pérez de la Torre et al., 2018). The rationale has often been to develop genetically stable compact plants as a bio-sustainable alternative to chemical growth retardants. Several places, e.g., Europe, the application of chemical growth retardants is increasingly restricted by legislation and as a result many of the most effective regulators have already been banned and further restrictions are expected (Rademacher, 2016). These restrictions have been implemented as several of those compounds exhibit potential detrimental effects on both the environment and human health (Rademacher, 2000, 2016; De Castro et al., 2004). However, numerous abiotic bio-friendly alternatives have been developed and are commonly applied in ornamental plant production, e.g., brushing and shaking of plants, cold-morning treatment, and light control (McMahon, 1999; Clifford et al., 2004; Bergstrand, 2017). Unfortunately, these methods do not completely fulfill the desired requirements for compact habit and reduced plant growth as the treatment effect ceases to work once the plant is out of the production site. Moreover, several efficient genetic engineering approaches exist to create stable compact phenotypes. These approaches have been pursued in several ornamental plants, e.g., *Calibrachoa* (Gennarelli et al., 2009), *Kalanchoë* (Christensen et al., 2008), *Mecardonia* (Pérez de la Torre et al., 2018), *Petunia* (Mishiba et al., 2006), and poinsettia (Islam et al., 2013). Some of these strategies target alteration of gibberellic acid (GA) metabolism; GA_20_ oxidases have silenced (Topp et al., 2008), GA_2_ oxidases have been over-expressed (Gargul et al., 2013), the *Arabidopsis thaliana Short Internodes* gene has been overexpressed (Lütken et al., 2010; Islam et al., 2013), and knotted homeobox genes have been modulated (Lütken et al., 2011). Although being successful, these strategies unfortunately all fall under the EU GMO regulation (European Union, 2001) and are, due to legislation and low public acceptance, presently not applicable for the horticultural market in e.g., Europe. In this respect, transformation with unmodified bacterial strains of e.g., *R. rhizogenes* without the use of recombinant DNA technologies and the plants derived from this approach are not classified as GMO according to the current European GMO legislation (European Union, 2001). The terminology of “natural transformation” has been used as a label for these strategies and it presents a very promising beacon for generating compact habit and reduced plant growth (as reviewed by Lütken et al., 2012a; Desmet et al., 2019).

Understanding how *R. rhizogenes* confers compact habit and reduced growth traits in plants is highly relevant given the potential to reduce the use of chemical growth retardants. When plants are infected with *R. rhizogenes*, T-DNA from the root inducing (Ri)-plasmid is inserted and integrated in the plant host DNA (Chilton et al., 1982) leading to the development of the Ri-phenotype, in which hairy roots protrude from the site of infection (Tepfer, 1990). The Ri-plasmid of agropine strains is a split T-DNA plasmid, harboring two regions designated as the left T-DNA (T_L_-DNA) and right T-DNA (T_R_-DNA), which are separated by about 15–20 kb of non-transferred DNA (White et al., 1985). Numerous genes and open reading frames (ORFs) are present on the T_L_-DNA. The most well-known and characterized genes known to be involved in the compact habit and reduced plant growth, are the *root oncogenic loci* (*rol*) genes; *rol*A, *rol*B, *rol*C, and *rol*D, which correspond to ORFs 10, 11, 12, and 15, respectively (White et al., 1985; Slightom et al., 1986; reviewed by Rangslang et al., 2018; De Paolis et al., 2019). In comparison, the T_R_-DNA harbors *aux1* and *aux2* genes (*iaaM* and *iaaH*, respectively; Camilleri and Jouanin, 1991) involved in auxin biosynthesis (Mashiguchi et al., 2019) as well as a homolog of the *rol*B gene, termed *rol*BTr (Bouchez and Camilleri, 1990). Plants can be regenerated from the hairy roots following treatment with various, often high, and ratios of cytokinins:auxins (Hegelund et al., 2017; Mandal et al., 2020; Neumann et al., 2020). Regenerated plants exhibit, among other features, compact habit, reduced plant growth, and frequently thicker leaves that are often reduced in size (as reviewed by Lütken et al., 2012a; Rangslang et al., 2018).

In attempts to unravel the effect of the individual *rol* genes, several genetic constructs with individual as well as multiple *rol* genes/ORFs delivered using *Agrobacterium tumefaciens* have been investigated in a range of plant species. Analysis of especially *rol*A–D, but also some of the other ORFs has indicated that several of these genes/ORFs influence plant morphology and hormone sensitivity specifically as well as differently (Casanova et al., 2005). Presence of *rol*A typically leads to wrinkled leaves (Sinkar et al., 1988; Bettini et al., 2016b). It has been demonstrated that *rol*B has a crucial role in hairy root formation. In tobacco, its expression alone is sufficient to produce roots that are often fast growing, highly branched, and ageotropic (Cardarelli et al., 1987). Additionally, *rol*B plants display necrosis in leaves and altered shoot morphology, including increased flower size and change in leaf shape (Schmulling et al., 1989; Kodahl et al., 2016). Plants transformed with *rol*C under its endogenous promoter display dwarf phenotypes with reduced apical dominance, lanceolate leaves, early inflorescence, and smaller flowers (Schmülling et al., 1988). It has been speculated that *rol*C increases the levels of active cytokinins, based on the findings that its encoded protein has beta-glucosidase activity which enables the release of free active cytokinins from their active conjugates; observed *in vitro* (Estruch et al., 1991a). Although being the least studied *rol*-gene, *rol*D is the only gene where the protein function is elucidated; it encodes an ornithine cyclodeaminase (Trovato et al., 2001). In respect to the ORFs, e.g., ORF13, ORF13a, and ORF14, less information is available. In transgenic tobacco plants harboring ORF13, phenotypic alterations were detected, such as dwarfing, wrinkled leaves, and shortened internodes (Lemcke and Schmülling, 1998). Interestingly, it was recently found that ORF13 confers a major role in the compact habit phenotype when overexpressed in *Arabidopsis* (Kodahl et al., 2016). The ORF located on the lagging strand of the Ri plasmid between ORF13 and ORF14 was termed ORF13a (Hansen et al., 1991) and is speculated to encode a regulatory protein (Hansen et al., 1994). Overexpression of ORF13a in *Nicotiana tabacum* did not exhibit phenotypic effects in the regenerated F1 populations (Lemcke and Schmülling, 1998). For ORF14, it was found that overexpression has no changes in the transgenic plant’s morphology (Lemcke and Schmülling, 1998). It has been suggested that ORF13 and ORF14 co-act in synergy with the other *rol* genes, improving root induction in *N. tabacum* and *Daucus carota* (Capone et al., 1989; Aoki and Syono, 1999a).

In the economically important *Kalanchoë*, many studies have been made to investigate the effects of the inserted T-DNA of *R. rhizogenes* (Lütken et al., 2012a), and several transformants exhibited large variations in plant diameter, number of branches, flower diameter, time to first open flower, and duration of flowering compared to control plants (Christensen et al., 2010). Advanced knowledge on the specific phenotype effects of the individual *rol* genes and ORFs transferred *via A. tumefaciens* will be beneficial to guide back-crossing approaches to confer compactness from naturally *R. rhizogenes* transformed plants. Moreover, detailed molecular data and more specifically the protein sub-cellular localization using transient expression *via A. tumefaciens* could prove beneficial in future characterization of encoded proteins of *rol* genes/ORFs.

This study aimed to investigate the phenotypic effects of individual *rol* genes/ORFs on regenerated plants of *Kalanchoë blossfeldiana*. This was obtained by producing and characterizing transgenic lines with 35S driven overexpressing cassettes separately containing *rol*B, *rol*C, ΔORF13a, and ORF14 with special focus on postharvest aspects such as ethylene tolerance in detached flowers. Moreover, relative copy number and gene expression data were obtained. In addition, due to the strong phenotypes observed for *rol*C and ΔORF13a transformed lines, we investigated the subcellular localization of their encoded proteins to provide deeper molecular understanding of *rol* genes/ORFs.

## Materials and Methods

### Annotation of ORF13a From pRiA4 and Gene Cloning

The reported sequence for the T_L_-DNA (Genbank accession: K03313) of the Ri plasmid of *R. rhizogenes* A4 strain (ATCC43057) was annotated using the FgenesH tool (Solovyev et al., 2006). Subsequently, a previously annotated sequence of ORF13a from pRi8196 (Genbank accession: AAA22098; Hansen et al., 1991) was used to search for the corresponding homologous ORF13a from the predicted ORFs by FgenesH. Sequences were compared by using BLASTp. Primers for cloning a truncated ORF13a (ΔORF13a) sequence from *R. rhizogenes* A4 strain were specifically designed to exclude the putative regulatory region with the SPXX motif repeats (Supplementary Table S1).

Selected genes and ORFs from *R. rhizogenes* A4, i.e., *rol*B, *rol*C (Genbank accession number: MT514512), ΔORF13a (Genbank accession: MT514511), and ORF14, were previously available in pDONR™221 and recombined into the destination vector pK2GW7 (Karimi et al., 2002) using the Gateway™ LR Clonase™ II Enzyme mix (Invitrogen, Carlsbad, CA, United States), according to Kodahl et al. (2016). The destination vector includes selection markers streptomycin/spectinomycin and kanamycin for bacteria and plants, respectively, as well as the constitutive plant promoter *35S* derived from *Cauliflower mosaic* virus. All PCR-based constructs were verified by Sanger sequencing (Mix2Seq kit, Eurofins Genomics, Ebersberg, Germany; Supplementary Tables S1, S2). The sequencing verified vectors were transformed by electroporation (MicroPulser Electroporator, Bio-Rad) into rifampicin resistant and competent *A. tumefaciens* C58C1 (pGV3850). Colony selection was performed in *Agrobacterium* growth media supplemented with 10 g L^−1^ agar (Duchefa Biochemie B.V., Haarlem, The Netherlands), followed by single-colony inoculated liquid culture maintained in *Agrobacterium* growth media (Supplementary Table S3). Additionally, successful *A. tumefaciens* transformation was verified by plasmid purification (GenElute™ Plasmid Miniprep Kit, Sigma-Aldrich, St. Louis, MO, United States) followed by PCR detection of the inserted target gene using full-length gene specific primers (Kodahl et al., 2016).

### Plant Material

Vegetatively propagated *K. blossfeldiana* ‘Molly’ plants were grown in 10 cm diameter pots with sand, vermiculite, and Pindstrup peat substrate in a 1:1:1 ratio (Substrate no. 1, PindstrupMosebrug A/S, Ryomgard, Denmark), saturated with a solution of 1 ml L^−1^ Gnatrol® (Valent BioSciences, Libertyville, IL, United States), and kept in long day greenhouse conditions (Supplementary Table S4). The watering was based on substrate drying assessment, i.e., approximately 2–3 per week, utilizing ebb/flow irrigation. The fertilized water contained nitrogen, phosphorus, and potassium supplemented with magnesium (1 g L^−1^ of Pioner NPK Makro Blå 14-3-23+[3]Mg, Azelis Denmark A/S, Kgs Lyngby, Denmark) and micronutrients (0.1 ml L^−1^ of Pioner Mikro med Jern, Azelis Denmark A/S), adjusted to pH 6.0 and with electrical conductance of 2.0 μS cm^−1^. Leaf material was selected for transformation from up to 3-month old plants, collected, and surface-sterilized using 70% ethanol (VWR chemicals, Søborg, Denmark) for 1 min, followed by 90 g L^−1^ Ca(ClO)_2_ (ACROS Organics, Geel, Belgium) and 0.03% Tween® 20 (Sigma-Aldrich) for 20 min and rinsed three times in sterile deionized water. Additionally, the leaves were cut into 1 cm^2^ pieces, excluding the leaf margins and central vein, and the generated explants were placed in sterile deionized water until inoculation according to Lütken et al. (2010).

### *Kalanchoë blossfeldiana* Transformation and *de novo* Organogenesis

Pre-inoculum liquid cultures of *A. tumefaciens* were initiated by adding 1 ml glycerol stock for each construct to 10 ml *Agrobacterium* growth media (Supplementary Table S3) and cultured O/N at 28°C and 200 rpm (Topp et al., 2008). The inoculum was prepared by increasing the liquid culture volume to 200 ml with *Agrobacterium* growth media and subsequently, adjusting the optical density (OD) to 0.2 using the inoculation media (Supplementary Table S3) as diluent and cultured for 2–4 h at 28°C and 200 rpm. Prior to inoculation, the OD was measured again and adjusted to 0.4–0.6 with inoculation media. *Kalanchoë* leaf explants were inoculated with the adjusted *A. tumefaciens* liquid cultures according to Lütken et al. (2010). The experimental control included plant material submerged in inoculation media without bacteria following the same criteria. Succeeding the co-cultivation period, the explants were immersed in washing media (Supplementary Table S3) containing antibiotics for 30 min under 50 rpm agitation, blotted, and transferred to *de novo* organogenesis media (Supplementary Table S3). *Kalanchoë* explants on *de novo* organogenesis media were placed at long day tissue culture growth conditions (Supplementary Table S4) for 4–6 months and sub-cultured to fresh media every 3–4 weeks until the appearance of shoots. Unrooted *Kalanchoë* shoots were excised from the callus material, transferred to rooting media (Supplementary Table S3) until the formation of roots, and placed at the same growth conditions and sub-culturing frequency. Upon significant rooting, e.g., >5 mm long roots, the plantlets were transferred to 10 cm diameter pots with Pindstrup peat substrate (Substrate no. 1, PindstrupMosebrug A/S) and saturated with a solution of Gnatrol® (Valent BioSciences). The pots were placed in the long day climate chamber (PGV56, Conviron, Winnipeg, MB, Canada) growth condition (Supplementary Table S4) for 4–6 weeks, i.e., intermediary between tissue culture and greenhouse conditions. The plantlets were covered with a plastic lid during the initial week to accommodate acclimation. The initial shoots and later plantlets remained green after successive tissue culture sub-cultivation rounds on media containing kanamycin, as well as showed stable phenotypes after at least three clonal propagations in the pots.

### Transgene Verification

Plant material, i.e., leaves, were collected, snap frozen, and pulverized using liquid N_2_, followed by −80°C storage. Genomic DNA extraction was proceeded with Plant DNA Isolation Reagent (Takara Bio Inc.) according to the manufacturer’s instructions and the concentration and purity assessed using a NanoDrop (ThermoFisher Scientific, Waltham, MA, United States). Genotyping was performed using specific primer sets targeting full-length *rol* genes/ORFs and *Kalanchoë daigremontiana Actin* (*KdActin*, control; Supplementary Table S1) were used. Multiple PCRs to amplify these products were performed using Ex Taq® DNA Polymerase (Takara Bio Inc.) as per supplier’s instructions and 40–100 ng gDNA added in each 25 μl reaction. The reactions were incubated in a DNA thermal cycler (MyCycler, Bio-Rad) following the programs listed in the Supplementary Table S2. The PCR products were mixed with GelRed (Biotium, Hayward, CA, United States), resolved in 1x TAE (ThermoFisher Scientific) 1.5% agarose (VWR chemicals) gel electrophoresis at 100 V for 55 min and visualized under UV-light (GelDoc XR+, Bio-Rad). Positive lines for the respective cassette were further clonally propagated. To attain that the correct gene/ORF sequence was successfully transferred, the selected lines for phenotyping and ethylene experiments were further assessed with a second PCR using the primer set *pK2GW7_35S_Fw1* and *pK2GW7_RB_Rv1* (Supplementary Table S1). Upon confirmation of a successful PCR amplification by gel electrophoresis, the purified PCR product (GeneJET PCR Purification Kit, ThermoFisher Scientific) was sent for Sanger sequencing (Mix2Seq kit, Eurofins Genomics) in both forward and reverse direction with the primers *pK2GW7_35S_Fw2* and *pK2GW7_T35S_Rv2* (Supplementary Table S1), respectively. An alignment (CLC Main Workbench, Qiagen) with no mismatches from the sequencing results to the expected sequence was assessed as the line being a true positive.

### Phenotypical Characterization

To evaluate and analyze the phenotypical differences between the transformed plants and wild type (WT) plants, the available positive lines from each gene construct plus one WT were selected and propagated. About 4–10 cuttings per positive line and eight WT were harvested, made uniform at 2–3 cm height, and planted into new pots filled with Pindstrup peat (Substrate no. 1, Pindstrup Mosebrug A/S) saturated with Gnatrol® solution (Valent BioSciences). Plants were initially grown in long day greenhouse condition (Supplementary Table S4) until they had developed three pairs of fully expanded leaves. Plants were then flower induced in short day greenhouse condition (Supplementary Table S4) corresponding to the same settings to produce commercial *Kalanchoë* (Lütken et al., 2012b). Irrigation and fertilization were as above mentioned. Data were collected monthly for plants’ biometrical characteristics, including plant height, plant diameter, and number of leaves and branches. The experiment was repeated twice displaced in time (4–6 weeks apart), and a total of four monthly measurements were done for each propagation. Data were also collected at the endpoint, consisting of a destructive analysis of the plants, i.e., total number of flowers, flower diameter and shape, leaf area (LI-3100 Area meter, Lincon, NE, United States), fresh and dry weight, and chlorophyll content (CCM-200, Opti-Science, Hudson, NH, United States) was evaluated on third and fourth-youngest leaf pairs.

### Relative Copy Number and Gene Expression of the Transgenic Lines

Relative copy number was determined by real-time quantitative PCR (RT-qPCR) on gDNA, and the reactions were performed on a ICycler instrument (CFX Connect Real-Time PCR Detection System, Bio-Rad) using SsoAdvanced™ Universal SYBR® Green Supermix (Bio-Rad) according to supplier’s instructions. Forty nanogram of gDNA was used in each 20 μl reaction. The following fragment primer pairs for *rol*B, *rol*C, ΔORF13a, and ORF14 at 200 nM final concentration were used correspondingly to the transgenic lines analyzed with *K. laxiflora Actin* (*KlActin*) as reference gene (Supplementary Table S1). Additionally, gDNA samples from naturally transformed *K. blossfeldiana* lines, namely 306, 324, and 331 were used as reference for known copy number based on Southern blot (Christensen et al., 2008). The following program was used: 98°C for 3 min, 45 cycles of (15 s at 98°C, 30 s at 60°C). Following each run, a melt curve analysis was done in the range 55–95°C with 0.5°C increments in 10 s per step. The threshold cycles (Ct) for these primer pairs were standardized using the corresponding *KlActin* Ct (ΔCt) and line 306 was used as reference for single copy (ΔΔCt). The relative quantification of the target genes, i.e., *rol*B, *rol*C, ORF13a, and ORF14, among different lines was determined as 2^−ΔΔCq^ using the CFX Maestro Software (Bio-Rad). Line 306 was arbitrarily defined as reference for 1-fold, due to one copy in the Southern blot, and relative distribution categories were based on Bartlett et al. (2008) and Lütken et al. (2010). Based on the samples with known copy number and their obtained fold change, the lines were categorized into the following significantly different groups: single copy (fold change < 2), low copy number (2 > fold change < 15), and high copy number (fold change > 15). Values are based on three replicates and were repeated twice.

Total leaf RNA was isolated from a pooled sample of three plants of the same line using RNeasy Plant Mini Kit (Qiagen) and digested with RNAse-free DNAse (Sigma-Aldrich) following the manufacturer’s protocol with minor modification. RLC buffer mixed with 30 μl 50% (v/v) PEG 20.000 (Sigma-Aldrich) per extraction was used for the plant cell lysis step. RNA yield and purity were determined by NanoDrop (ThermoFisher Scientific), and integrity was evaluated on 1% agarose (VWR chemicals) 1x TAE (ThermoFisher Scientific) supplemented with 1% sodium hypochlorite (VWR chemicals; Aranda et al., 2012). Deoxyribonuclease I (Sigma-Aldrich) was used to remove residual DNA following the manufacturer’s instructions. Reverse transcription synthesis was performed on 0.8 μg DNase treated RNA using iScript cDNA Synthesis Kit (Bio-Rad) according to the manufacturer’s recommendations. The RT-qPCR used 400 ng of cDNA per 20 μl reaction as template mixed with SsoAdvanced™ Universal SYBR® Green Supermix (Bio-Rad) according to supplier’s instructions and incubated in the ICycler instrument (Bio-Rad). Each transgenic line was assessed with the corresponding above mentioned fragment primer pair and *KlActin* was the reference gene (Supplementary Table S1). The following program was used: 95°C for 30 s, 45 cycles of (95°C for 15 s, 60°C for 30s). A melt curve analysis was conducted in the range 55–95°C with 0.5°C increments in 10 s per step after each run. The threshold cycles (Ct) were standardized using the corresponding *KlActin* Ct (ΔCt). The relative quantification of the target genes, i.e., *rol*B, *rol*C, ORF13a, and ORF14, among different lines was determined as 2^−ΔCq^ using the CFX Maestro Software (Bio-Rad). Values are based on three replicates and were repeated twice.

### Tolerance of Detached Flowers to Exogenous Ethylene

Selected overexpressing lines, i.e. *rol*B, ΔORF13a, and ORF14, and WT control were propagated as previously described, being initially kept in long day greenhouse conditions until three pairs of fully opened leaves were reached and then transferred to growth chambers (PGV56, Conviron) programmed to short day conditions for flower induction (Supplementary Table S4). Irrigation and fertilization were as above mentioned. It was ensured that all flowers used in the experiment were 1–4 day old by removing all open flowers from the plants accordingly prior to experimental setup. Single open flowers were excised and placed in micro titer plates containing tap water according to Christensen and Müller (2009). The micro titer plates with flowers were transferred to glass tanks that were sealed and exposed to 0.5 μl L^−1^ ethylene (1,000 μl L^−1^ ethylene in N_2_, Calgaz, Staffordshire, United Kingdom) or air (control) for 5 days. The glass tanks were kept at ethylene growth chamber conditions (Supplementary Table S4). Ethylene tolerance was measured in terms of flower closure by daily recording the flower diameter *via* photographs (Iphone 9 plus, Apple, Cupertino, CA, United States) later analyzed in ImageJ (Rueden et al., 2017), and flower senescence was defined when the petals turned black and the flower was completely closed. Eight replicate flowers obtained from each overexpressing line and WT were considered a repetition. The experiment was repeated four times with flowers derived from two independent propagation rounds. The flower closure was calculated as percentage of the initial diameter of exposed flowers and flower senescence is displayed as percentage from the total assessed flowers.

### Sub-Cellular Localization of ΔORF13a and rolC in *Nicotiana benthamiana* Leaves

The preparation of monomeric Red Fluorescent Protein1 (mRFP1) and SbCYP98A1-mRFP1 constructs was conducted as described previously (Laursen et al., 2016). The construction of *rol*C and ΔORF13a fused to enhanced Green Fluorescent Protein (eGFP) at the C or N terminus was done by amplifying the full-length coding sequences with appropriate primers (Supplementary Table S1), and the amplicons were inserted in the pCAMBIA1300/UeGFP or pCAMBIA1300/eGFPU vectors, respectively, by the single-insert USER cloning technique (Geu-flores et al., 2007). All PCR-based constructs were verified by sequencing. Expression constructs were transformed into *A. tumefaciens* strain AGL1. Colonies of *A. tumefaciens* were picked and pre-cultured in 3 ml of LB medium with appropriate antibiotics. Afterward, 10 ml of LB medium containing antibiotics were inoculated with 50 μl of the pre-culture and incubated at 28°C overnight or until reaching an OD_600_ of 1.5. The cultures were centrifuged, the cell pellet was resuspended in infiltration buffer (10 mM MgCl_2_, 10 mM MES, pH 5.6, and 100 μM acetosyringone), and OD_600_ was adjusted to 0.15. *A. tumefaciens* strains carrying the constructs of interest were co-infiltrated in equal densities with *A. tumefaciens* transformed with a pCAMBIA1300 vector for expression of the viral p19 silencing suppressor protein (Lakatos et al., 2004). Leaf discs from transformed plants were sampled 3 days after infiltration for observation by confocal laser scanning microscopy. An SP5x confocal laser scanning microscopy device equipped with a DM6000 microscope (Leica, Wetzlar, Germany) was used to record images of enzyme subcellular localization with settings described by Laursen et al. (2016).

### Statistics

The experiments were conducted in complete randomized design, repeated at least twice over time and ANOVA was performed using the software Sisvar (Ferreira, 2011), considering the lines, i.e., WT, *rol*B1, *rol*B2, *rol*C1, ΔORF13a, and ORF14 as source of variation. The data with *p* ≤ 0.05 in the F-test followed the Scott Knott average comparison test (*p* ≤ 0.05). Relative gene expression data were compared using Student’s *t*-test (Student, 1908). Data are presented as average ± SE (*n* = 6–32).

## Results

### Sequences of ORF13a Differs Between Two *Rhizobium rhizogenes* Strains

The ORFs from the T_L_-DNA sequence of the pRiA4 (agropine type) reported by Slightom et al. (1986) that would be transcribed in plants were predicted by the FgenesH tool. The sequence of ORF13a was found by using blastn and blastp algorithms with the nucleotide and amino acid sequence of ORF13a from pRi8196 (mannopine type) previously reported by Hansen et al. (1991). The ORF predicted by FgenesH with the highest similarity was ORF10 resulting from the algorithm and was 228 nucleotides long from position 15,012–14,785 in the reverse strand of the pRiA4 and consisted of one exon. The nucleotide sequence for the ΔORF13a of pRiA4 was published in Genbank with the following accession number: MT514511. Comparison of the ORF13a amino acid sequence from the agropine pRiA4 and the mannopine pRi8196 by blastp revealed significant difference in this ORF among the strains. Albeit sequences shared 68% identity and 76% similarity at amino acid level, the ORF13a sequence from pRiA4 is 34 amino acids shorter than that of pRi8196; accordingly, the pRiA4 ORF13a sequence starts at the amino acid 36 of the ORF13a of pRi8196 and the alignment continues to the end of both sequences. Thus, the putative regulatory region with the SPXX motif repeats (which corresponds to four amino acid residues Ser-Pro-X-X) in the ORF13a of pRi8196 is also present in the pRiA4 ORF13a, but with one SPXX motif missing. The region with SPXX motif repeats was excluded for two reasons: (1) although SPXX repeats could function in DNA-binding they could also be under regulation by kinases (Liu et al., 2018) and (2) Lemcke and Schmülling (1998) already overexpressed the full-length ORF13a from pRiA4 in *N. tabacum* and did not observe a distinct phenotype.

### Transformation Efficiencies of *K. blossfeldiana* Vary for the Individual *rol* Genes and ORFs

**Figure 1:**
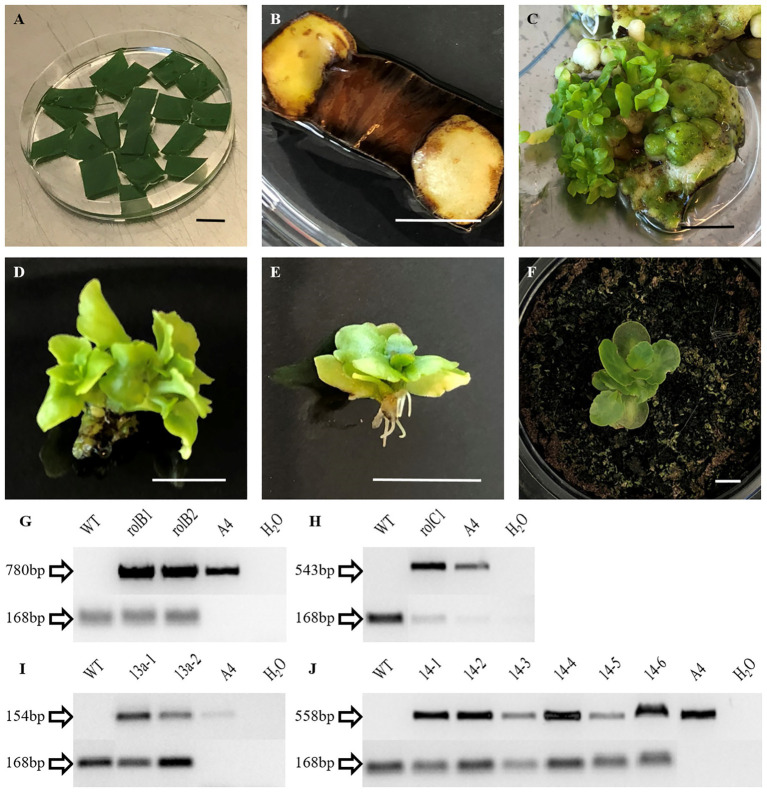
Transformation, regeneration, and molecular characterization for *Kalanchoë blossfeldiana* ‘Molly’. *K. blossfeldiana* ‘Molly’ **(A)** surface sterilized explants ready for inoculation **(B)** senescence associated with kanamycin selection. **(C)** Callus and initial shoots in *K. blossfeldiana* ‘Molly’ explants **(D)** excised shoot, and **(E)** at a later stage bearing roots. **(F)** *Kalanchoë blossfeldiana* ‘Molly’ rooted shoots transferred to peat. Genotyping by PCR of regenerated and selected *K. blossfeldiana* ‘Molly’ lines using full-length primers targeting **(G)** *rol*B (780 bp), **(H)** *rol*C (543 bp), **(I)** ΔORF13a (154 bp), and **(J)** ORF14 (558 bp). *Kalanchoë daigremontiana Actin* (*KdActin*) was the plant reference gene with amplicon of 168 bp, A4 is the unmodified plasmid from *Rhizobium rhizogenes* and positive control for the *rol* genes/open reading frames (ORFs) PCR reactions, and H_2_O was the negative control for both plant and *rol* genes/ORFs PCR reactions (see Supplementary Table S1 for the detailed list of primers). WT, wild type. Bar = 1 cm.

Leaf explants of *K. blossfeldiana* ‘Molly’ (Figure 1A) were successfully transformed with *rol*B, *rol*C, ΔORF13a, and ORF14 cassettes derived from *R. rhizogenes* A4. The kanamycin resistant shoots of each construct were verified by PCR and confirmed by sequencing; the transformation efficiencies ranged between 0.5 and 2.9% (Table 1). Kanamycin selection was effective (Figure 1B) and *de novo* organogenesis was obtained by media containing high cytokinin:auxin levels of plant growth regulators (see *K. blossfeldiana* Transformation and *de novo* Organogenesis section), which yielded in callus (Figure 1C), shoot (Figure 1D), and root (Figure 1E) formation that enabled the generated plantlets to be transferred to pots (Figure 1F). The presence of the T-DNA bearing the overexpressing constructs was confirmed by PCR amplification (Figures 1G–J) and followed by sequencing (data not shown).

**Table 1:** Overall *Agrobacterium tumefaciens* transformation data. Number of inoculated explants, kanamycin resistant shoots and confirmed by PCR amplicon sequencing, and transformation efficiency for the investigated constructs.

Construct	No. of explants	No. of kanamycin resistant shoots	No. of PCR-positive shoots	Transformation efficiency (%)
*rol*B	201	3	2	1.0
*rol*C	201	1	1	0.5
ΔORF13a	274	6	3	1.1
ORF14	272	13	8	2.9

### Compact Habit and Reduced Plant Growth Was Achieved to Different Extent in *K. blossfeldiana* Transformed With *rol* Genes and ORFs

**Figure 2:**
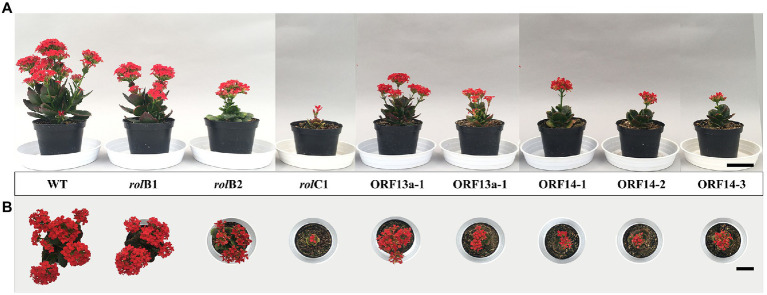
Phenotype of transgenic lines. **(A)** Frontal and **(B)** top view of transgenic lines and wild type (WT) *K. blossfeldiana* ‘Molly’ plants at 4 months after the flower induction, i.e. short day treatment, was initiated. Bar = 5 cm.

**Figure 3:**
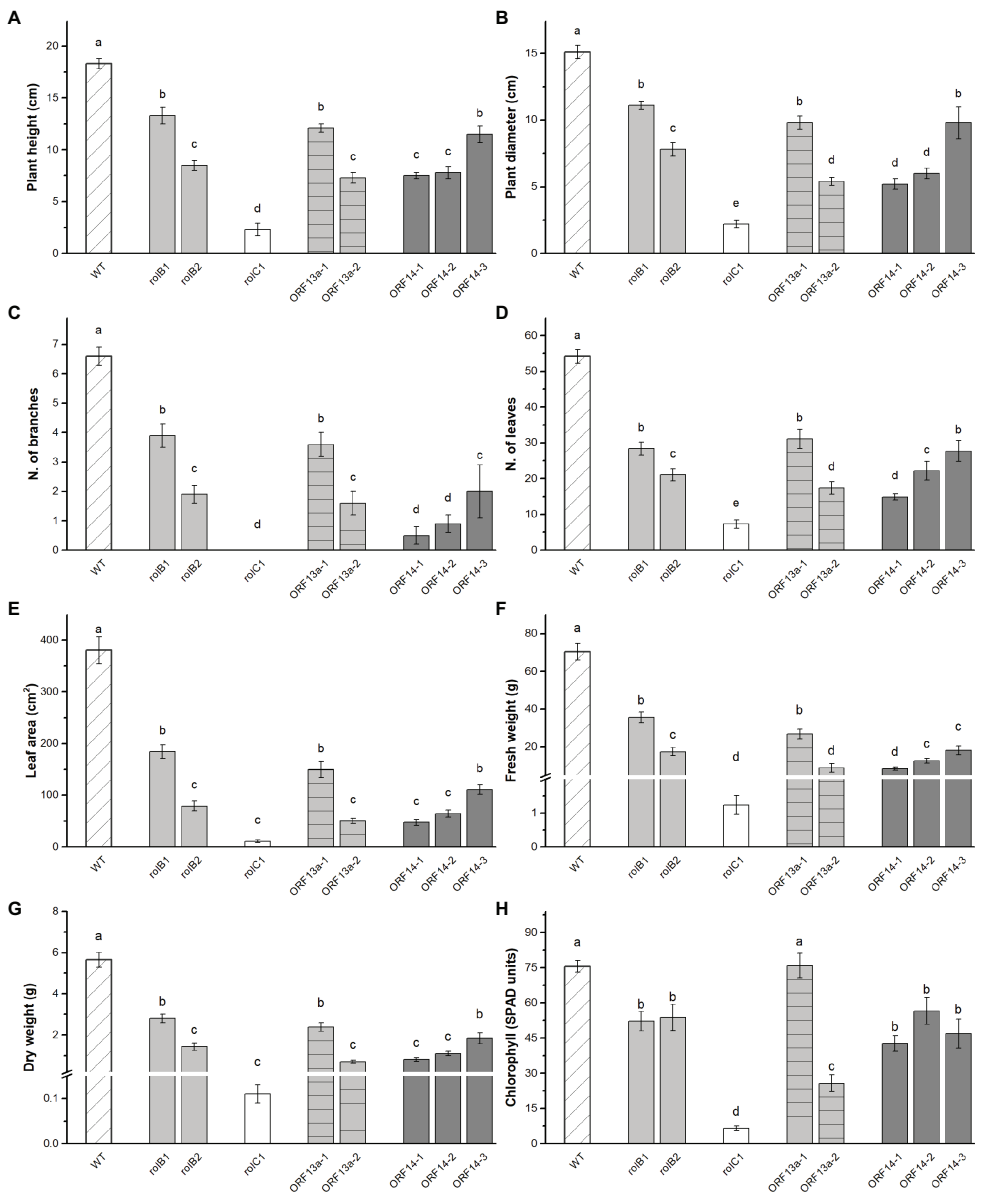
Vegetative biometric data and chlorophyll content in leaves of plants derived from individual transformation events. **(A)** Plant height, **(B)** plant diameter, **(C)** number of branches, **(D)** number of leaves, **(E)** leaf area, **(F)** fresh weight, **(G)** dry weight, and **(H)** chlorophyll of WT *K. blossfeldiana* ‘Molly’ and overexpressing lines (*rol*B1, *rol*B2, *rol*C1, ΔORF13a-1, ΔORF13a-2, ORF14-1, ORF14-2, and ORF14-3). Data columns represent the average ± SE (*n* = 6–27) and different lower case letters represent statistical significance using Scott-Knot test (*p* ≤ 0.05).

The regenerated lines were clonally propagated in order to perform phenotyping analysis. It was observed that the plant height, diameter, leaf area, and fresh and dry weight of the *rol* genes/ORFs regenerated overexpressing lines were visually (Figures 2A,B) and statistically significant (Figures 3A–F) lower than the WT line. Height showed inter-line variation, i.e., *rol*B1 was 36% higher than *rol*B2, ΔORF13a-1 was 39% higher than ΔORF13a-2, and ORF14-3 was 33% higher than ORF14-1 and ORF14-2 (Figure 3A). Moreover, all these transgenic lines were statistically significant shorter than the WT (18.3 ± 0.4 cm). These inter-line variations were also observed for the other investigated traits, i.e., diameter, number of branches, number of leaves, leaf area, and fresh and dry weight (Figures 3B–G). The number of leaves was negatively affected in the *rol* genes/ORFs overexpressing lines compared to WT (54.2 ± 1.9 leaves), the decrease was most pronounced in *rol*C1 (87%) and least in ΔORF13a-1 (43%; Figure 3D). Moreover, the leaf area was severely impaired by the presence of the overexpressing *rol* genes/ORFs cassettes in comparison to WT (Figure 3E). In contrast to previous observations, plant height and diameter (Figures 3A,B), ORF14-2 showed 12% more leaves than ORF14-1; however, the leaf area did not differ between the two lines (Figures 3C–E). In terms of biomass, the fresh and dry weight variation of the overexpressing lines fluctuated negatively in the same order of magnitude for both parameters when compared to WT. The least reduction was observed for *rol*B1 line displaying 49% decrease of leaves in relation to the WT (Figures 3F,G). In addition, assessment of the relative chlorophyll content indicated that the chlorophyll accumulation of *rol*B1, *rol*B2, and ORF14-1-3 lines decreased on average 27%, while 91 and 66% in the *rol*C1 and ΔORF13a-2 lines, respectively, when compared to WT (Figure 3H). In contrast, chlorophyll accumulation of ΔORF13a-1 was not altered compared to WT (75.6 ± 2.5 SPAD units).

**Figure 4:**
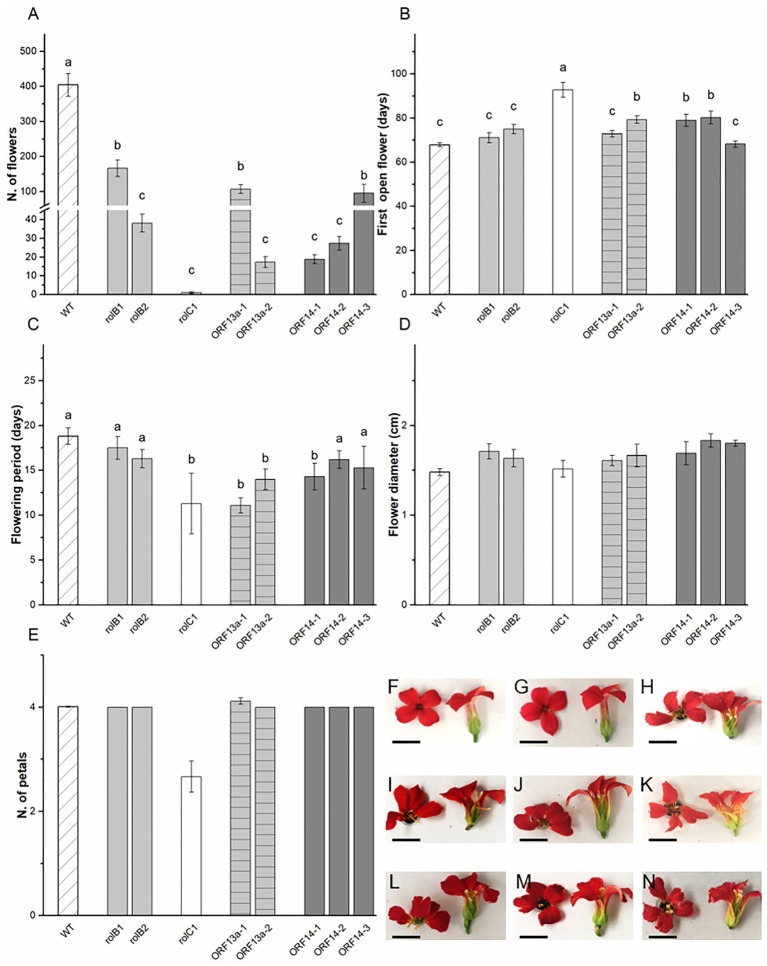
Generative biometric data and flower morphology. **(A)** Number of flowers, **(B)** days to first open flower, **(C)** flowering period, **(D)** flower diameter, and **(E)** number of petals of WT *K. blossfeldiana* ‘Molly’ and overexpressing lines (*rol*B1, *rol*B2, *rol*C1, ΔORF13a-1, ΔORF13a-2, ORF14-1, ORF14-2, and ORF14-3). Data columns represent the average ± SE (*n* = 6–27) and different lower case letters represent statistical significance using Scott-Knot test (*p* ≤ 0.05). Graphs **(E)** and **(F)** were not statistically significant. Representative flower of **(F)** WT, **(G)** *rol*B1, **(H)** *rol*B2, **(I)** *rol*C1, **(J)** ΔORF13a-1, **(K)** ΔORF13a-2, **(L)** ORF14-1, **(M)** ORF14-2, and **(N)** ORF14-3 illustrating morphological changes between the lines. Bar = 0.5 cm.

The biometric data for the ornamental features of the *K. blossfeldiana* ‘Molly’ transgenic lines, i.e., flowers, were collected throughout the period and at the end of the flower induction phase. Parameters regarding developmental, i.e., anthesis and postharvest longevity, and morphological, i.e., flower diameter and petal arrangement, features were assessed. The number of flowers in the WT was 404 ± 32 and severely decreased in the overexpressing lines, varying from 59% less flowers in *rol*B1 to 99% fewer flowers in *rol*C1 (Figure 4A). In terms of anthesis, the WT had its first open flower at 68.9 ± 0.9 days after the short-day treatment started with *rol*B1, *rol*B2, ΔORF13a-1, and ORF14-3 showing statistically similar timing, while 12 days later flowering was observed for ΔORF13a-2, ORF14-1, and ORF14-2 (Figure 4B). Postharvest longevity, defined as time from first open to first wilted flower, was equal or decreased for the transformed lines compared to WT (18.8 ± 0.9 days, Figure 4C). Flower diameter remained unchanged despite the presence of overexpressed *rol* genes/ORFs and alteration in the usual *Kalanchoë* 4-petal configuration was observed in the *rol*C1 line, although not significant due to the low number of observed flowers in this line (Figures 4D,E). The *rol*B1 flower (Figure 4G) had similar morphology as WT flowers presenting a fused tubular corolla with smooth edged petals (Figure 4F). However, morphological alterations were observed; i.e., unfused and fan shaped corolla in *rol*B2, *rol*C1, ΔORF13a-1, ΔORF13a-2, ORF14-1, and ORF14-3 (Figures 4H–L,N), irregular petal edges (lobose) in *rol*B2, *rol*C1, ORF14-2, and ORF14-3 (Figures 4H,L–N).

### Expression of *rol* Gene/ORF Transcripts Depended on the Number of Insertions

In total, two *rol*B, three ΔORF13a, and four ORF14 lines were analyzed for relative gene-copy number by quantitative real-time PCR according to methodology described by Lütken et al. (2010). The presence of the endogenous *KlActin* gene (Wang et al., 2019) was the reference used to normalize the RT-qPCR data, while the relative copy number of *rol* genes/ORFs were standardized using line 306 (Christensen et al., 2008) as reference for single copy, i.e., 1-fold change. Overall, a correlation to Christensen et al. (2008) Southern blot data was found, although in the current study *K. blossfeldiana* 300 lines did not entirely correlate when RT-qPCR was performed using *rol*C primers (Table 2). The following lines were attributed to have single copy insertions based on their fold change after RT-qPCR and in relation to the abovementioned categories: *rol*B2, ORF14-2, and ORF14-4; while these were tentatively classified as bearing low copy insertions: *rol*B1, ΔORF13a-1 and ΔORF13a-2, ORF14-1, and ORF14-3 (Table 2). The *rol*C1 line was originally verified for gene presence and confirmed by sequencing, but later the gene could not be detected in the mother plant anymore despite the consistent phenotype and was thus discarded from the analysis.

**Table 2:** Relative copy number of the *K. blossfeldiana* transgenic lines.

RT-qPCR target	Genotype	Fold change	Relative no. of copies
*rol*B	*rol*B1	5.9 ± 1.1	low copy
	*rol*B2	1.5 ± 0.2	single copy
	306	1.0 ± 0.1	single (1∗)
	331	11.1 ± 1.1	low copy (2∗)
	324	21.3 ± 1.2	high copy (9∗)
ΔORF13a	ΔORF13a-1	7.0 ± 1.2	low copy
	ΔORF13a-2	4.8 ± 0.7	low copy
	306	1.0 ± 0.1	single (1∗)
	331	10.3 ± 0.7	low copy (2∗)
	324	19.4 ± 1.6	high copy (9∗)
ORF14	ORF14-1	3.7 ± 1.5	low copy
	ORF14-2	1.7 ± 0.6	single copy
	ORF14-3	2.5 ± 0.9	low copy
	ORF14-4	0.4 ± 0.0	single copy
	306	1.0 ± 0.1	single (1∗)
	331	14.5 ± 2.4	low copy (2∗)
	324	25.0 ± 5.7	high copy (9∗)
*rol*C	306	1.0 ± 0.1	single (1∗)
	331	8.3 ± 1.0	low copy (2∗)
	324	10.3 ± 1.9	low copy (9∗)

The relative rol genes/ORFs copy number inserted into the genome was calculated in terms of fold change (2^−ΔΔCt^) standardized by *K. laxiflora Actin (*KlActin) and line 306. Categories were defined based on fold change: single copy (<2), low copy number (2 > and < 15), and high copy number (>15). ∗Data represent number of bands observed in Southern blot (Christensen et al., 2008). Data represent the average ± SE of three pooled plants for each line and the experiment was repeated twice.

The relative expression of *rol* genes/ORFs was assessed in leaves of transgenic *K. blossfeldiana* lines. RT-qPCR was conducted and the expression levels of the *rol* genes/ORFs lines were correlated to the expression of endogenous *KlActin* and WT plants. The highest relative expression level (correlated within the lines) was found in *rol*B2 (5.0 ± 0.8), ΔORF13a-2 (1.8 ± 0.1), and ORF14-2 (3.7 ± 0.5), all statistically significant. Whereas, the remaining of the lines displayed low relative expression levels. All transgenic lines exhibited expression of *rol* genes/ORFs to various levels (Table 3), except the *rol*C1 line, which was originally verified for gene presence and confirmed by sequencing, and displayed a consistently compact habit and reduced plant growth, but could not be further detected in the mother plant thus discarded from this analysis.

**Table 3:** Gene expression of the different *K. blossfeldiana* transgenic lines.

Genotype	Fold change
*rol*B1	0.1 ± 0.1b
*rol*B2	5.0 ± 0.8a
ΔORF13a-1	0.8 ± 0.2b
ΔORF13a-2	1.8 ± 0.1a
ORF14-1	2.5 ± 0.5b
ORF14-2	3.7 ± 0.5a
ORF14-3	2.5 ± 0.2b
ORF14-4	2.6 ± 1.2b

The relative rol genes/ORFs expression was standardized by KlActin and calculated in terms of fold change (2^−ΔCt^). Data represent the average ± SE of three pooled plants for each line, the experiment was repeated twice and different lower case letters within the rol gene/ORF represent statistical significance using *t*-test (*p* ≤ 0.05).

### Detached Flowers of Line ORF14-3 and ΔORF13a-1 Exhibited Changes in Ethylene Tolerance

**Figure 5:**
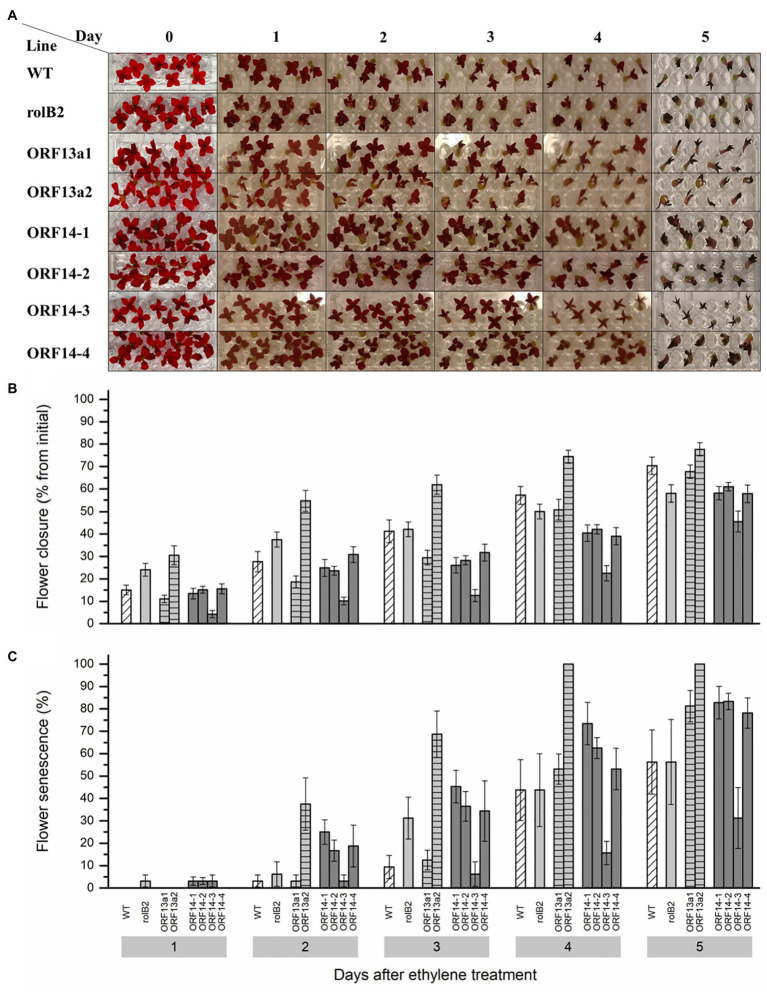
Ethylene sensitivity among the *rol* genes/ORFs overexpressing lines. **(A)** Exogenous ethylene (0.5 μl L^−1^) applied to detached *K. blossfeldiana* ‘Molly’ flowers of WT and overexpressing lines (*rol*B2, ΔORF13a-1, ΔORF13a-2, ORF14-1, ORF14-2, ORF14-3, and ORF14-4) placed in micro titer plates with water inside a hermetic sealed glass tank. A control group of the same genotype consisted of the same setting without the addition of ethylene. **(B)** Flower diameter was collected daily for 5 days after sealing the glass tanks and the flower closure is presented as percentage from the initial diameter of ethylene exposed flowers. **(C)** Senescent detached flower percentage on each evaluation day. Data columns represent the difference average ± SE (*n* = 8–10) and different lower case letters represent statistical significance using Scott-Knot test (*p* ≤ 0.05) within the time point.

The tolerance to exogenous ethylene was measured in detached flowers by assessing flower closure and flower senescence. Ethylene tolerance of detached flowers was assessed by exposure to 0.5 μl L^−1^ ethylene and the ORF14-3 line was markedly more tolerant to this hormone exhibiting reduced flower closure (Figure 5A). Detached flowers of the ORF14-3 line exhibited 66% less closure of individual flowers in the first 3 days after ethylene exposure compared to WT flowers (Figure 5B). The other ORF14 lines, i.e., 1, 2, and 4, and ΔORF13a-1 performed better in terms of ethylene tolerance on day 3 than the WT, but not to the same level as ORF14-3. Additionally, *rol*B2 showed similar ethylene tolerance as the WT on day 3. However, ΔORF13a-2 exhibited decreased ethylene tolerance, indicating specific *rol* genes/ORFs effects and highlighting the importance of ethylene tolerance studies as an attractive trait for *Kalanchoë* breeders. Petal blackening and complete flower closure were the criteria for labeling flower senescence. In ORF14-3, an increased postharvest quality duration with <20% senescent flowers until day 4 after ethylene exposure was observed (Figure 5C). In contrast, more than 50% of the flowers senesced in all other overexpressing lines and WT on day 4.

### rolC and ΔORF13a Are Soluble Proteins and Localize to the Cytoplasm

**Figure 6:**
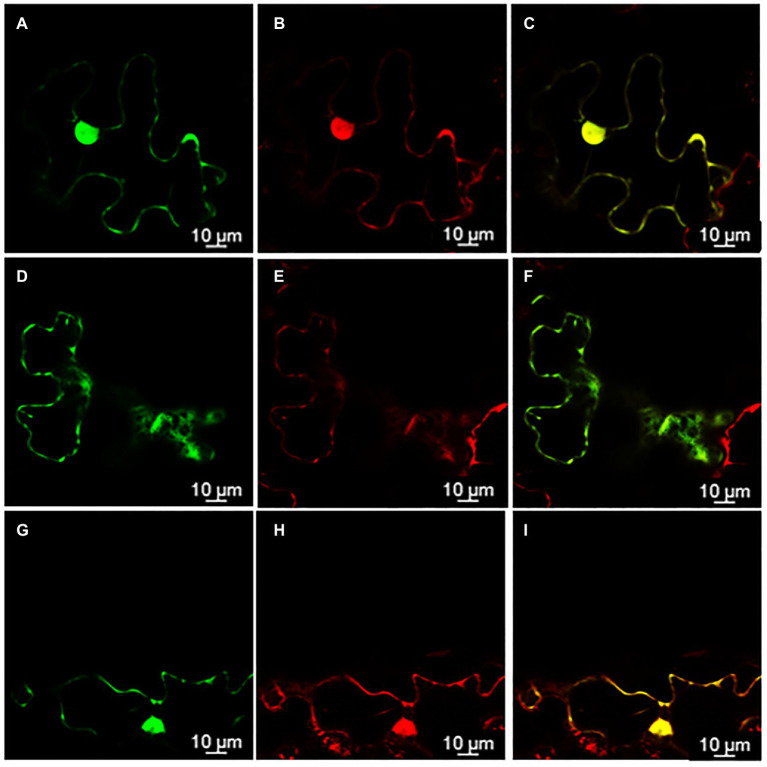
ΔORF13a localization in the cytoplasm visualized by confocal microscopy of *Nicotiana benthamiana* epidermal leaves. **(A)** ΔORF13a fused to enhanced Green Fluorescent Protein (eGFP) on its C-terminal end permeates through the nuclear pores and can be found inside the nucleus **(B)** as confirmed by soluble monomeric Red Fluorescent Protein1 (mRFP1) control, panel **(C)** is an overlay of signals from ΔORF13a-eGFP and mRFP1 demonstrating complete co-localization of both proteins. **(D)** Soluble proteins fill out the cytoplasm around the cortical endoplasmic reticulum (ER) as observed for ΔORF13a-eGFP and **(E)** mRFP1, panel **(F)** is an overlay of signals from ΔORF13a-eGFP and mRFP1 demonstrating complete co-localization of both proteins. **(G–I)** Fusion of eGFP to the N-terminal end of ΔORF13a did not affect the localization of ΔORF13a.

**Figure 7:**
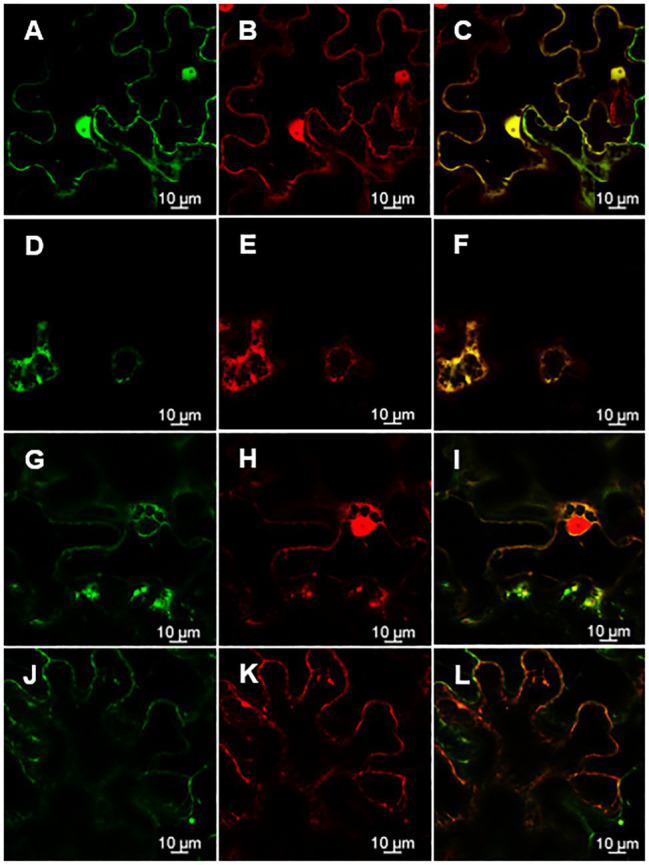
Position of the eGFP tag on rolC affects its subcellular localization as visualized by confocal microscopy of *N. benthamiana* epidermal leaves. **(A)** rolC fused to eGFP on its C-terminal end permeates through the nuclear pores and can be found inside the nucleus **(B)** as confirmed by soluble mRFP1 control, panel **(C)** is an overlay of signals from rolC-eGFP and mRFP1 demonstrating complete co-localization of both proteins. **(D)** Soluble proteins fill out the cytoplasm around the cortical ER as observed for rolC-eGFP and **(E)** mRFP1, panel **(F)** is an overlay of signals from rolC-eGFP and mRFP1 demonstrating complete co-localization of both proteins. **(G)** rolC fused to eGFP on its N-terminal end observed on the nuclear membrane. **(H)** Soluble mRFP1 permeates into the nucleus. Panel **(I)** shows incomplete co-localization of eGFP-rolC and mRFP1. **(J)** rolC fused to eGFP on its N-terminal end. **(K)** Soluble mRFP1. **(L)** Overlay of signals from eGFP-rolC and mRFP1.

**Figure 8:**
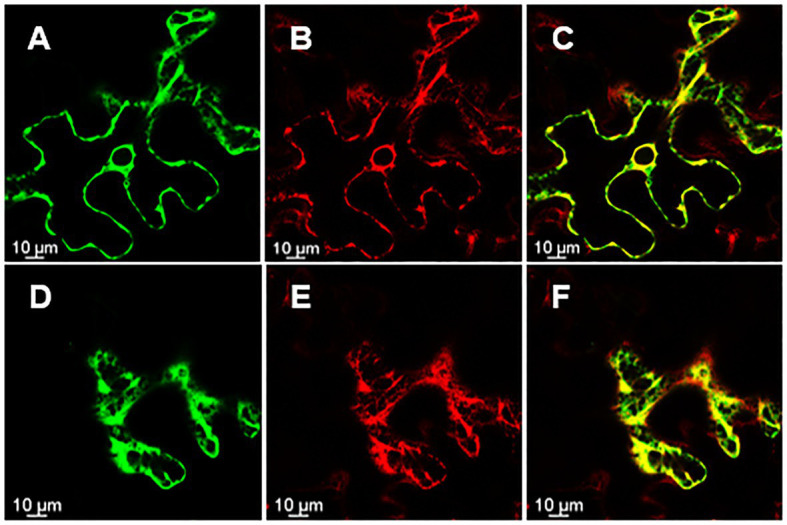
rolC with N-terminus eGFP tag visualized by confocal microscopy of *N. benthamiana* epidermal leaves. **(A)** rolC with eGFP tag on its N-terminal end localizes to the nuclear membrane **(B)** mRFP1 fused to SbCYP89A1. **(C)** Overlay of signals from eGFP-rolC and SbCYP89A1-mRFP1 demonstrating co-localization of both proteins on the nuclear membrane. **(D)** eGFP-rolC appears soluble filling the cytoplasm **(E)** Sb-CYP89A1-mRFP1 localizes to the ER membrane network; **(F)** is an overlay of signals from eGFP-rolC and SbCYP89A1-mRFP1 showing differential localization of both proteins.

Knowing the subcellular localization of a protein encoded by a gene may be useful when trying to elucidate the functional role of a gene. Thus, in order to assess the subcellular localization of *rol*C and ΔORF13a, constructs of their nucleotide sequence fused to eGFP at either the N or C terminus were prepared. Coexpression of the constructs with soluble mRFP1 as a control was performed in *Nicotiana benthamiana* to reveal the subcellular localization of the encoded proteins. Confocal images of ΔORF13a constructs tagged at both C and N termini showed that ΔORF13a is soluble and localizes in the cytoplasm, filling the space around the cortical endoplasmic reticulum (ER) as well as entering the nucleus (Figures 6A–I). The addition of the tag at either the N or C terminus did not influence the observed localization of ΔORF13a. In contrast, rolC tagged at the C terminus localized to the cytoplasm and entered the nucleus (Figures 7A–F). When rolC was tagged with eGFP at the N terminus, rolC appeared to be in the cytoplasm, but fluorescence was not detectable inside the nucleus, instead the fluorescence was only observed in the nuclear membrane (Figures 7G–L), suggesting that the eGFP tag interferes with the localization of the rolC protein. Therefore, to thoroughly assess the localization of rolC and possible effects of the eGFP tag, the localization experiments in *N. benthamina* were repeated coexpressing the *eGFP-rol*C construct with the construct of *SbCYP89A1* fused to *mRFP1* (as a control of a protein which localizes to ER and nuclear membrane). The confocal images only revealed overlay of the eGFP-rolC and SbCYP89A1-mRFP1 in the nuclear membrane, but not in the ER membrane network; eGFP-rolC remained a soluble protein localized to the cytoplasm, but was no longer detected inside the nucleus and remained at the nuclear membrane (Figures 8A–F).

## Discussion

In the current study, multiple transformation events generated independent transgenic lines of *rol*B, *rol*C, ΔORF13a, and ORF14 exhibiting clear morphological alterations. *De novo* organogenesis of *K. blossfeldiana* ‘Molly’ transgenic lines differed in efficiency between the investigated overexpression constructs, i.e., *rol*B, *rol*C, ΔORF13a, and ORF14, and the highest transformation rate was observed for ORF14 (2.9%) and lowest for *rol*C (0.5%). In this study, the transformation rate was lower than in earlier studies (Zia et al., 2010; Wang et al., 2019) and it followed previous transformation protocols optimized for *R. rhizogenes*-mediated transformation that included high concentration of cytokinin to promote the *de novo* organogenesis of shoots (Christensen et al., 2008; Hegelund et al., 2017). *Agrobacterium*-mediated transformation of multiple species of the *Kalanchoë* genus has been reported. For example, *K. laxiflora* transformed with either pCambia1305 or pCambia2305 in multiple *A. tumefaciens* strains revealed 4–21% positive transformation events *via* transient GUS activity assays (Wang et al., 2019). A compact habit and reduced plant growth was observed in all the transgenic lines when the overexpressing single gene assessment approach was utilized (Figures 2A,B). This is in accordance with other previous studies that targeted transformation with either WT *R. rhizogenes* (Christensen et al., 2008), single genes with endogenous promoters (Zhu et al., 2001; Bettini et al., 2003; Kubo et al., 2006), or with *35S* driven expression (Schmülling et al., 1988; Faiss et al., 1996; Koshita et al., 2002; Kodahl et al., 2016).

The transgenic lines of all investigated constructs displayed statistically significant (*p* ≤ 0.05) reduced plant height, diameter, number of branches, number of leaves, leaf area, fresh, and dry weight compared to WT (Figures 3A–G). In general, vegetative growth among the transformed plants changed greatly, this was the case for *rol*B1 and *rol*B2, which correlates to the observations in *A. thaliana* (Kodahl et al., 2016). In the present study, the *rol*B1 line was categorized as having a relative low copy number while *rol*B2 was estimated as a single copy line, and the expression levels in the latter had a 5-fold increase compared to *rol*B1. Therefore, a higher copy number indicated a lower expression of *rol*B. Similarly, Komarovská et al. (2010) observed that higher *rol* gene copy number integration was associated with weaker transgenic expression as well as a lesser aberrant phenotype in *Hypericum perforatum*. A stepwise reduction in plant height and diameter, leaf number, and area was observed for fresh and dry weight as well in decreasing order for WT > *rol*B1 > *rol*B2. Moreover, it seemed that in the *rol*B lines, a single copy event (Table 2) led to very high expression levels (Table 3), which in turn decreased all other phenotype parameters observed in this study (Figures 2A–G). This support the well-established paradigm of strong association of *rol*B to stunted growth and this has also been reported in *A. thaliana* (Dehio and Schell, 1994; Kodahl et al., 2016), *N. tabacum* (Schmülling et al., 1988), and *Lycopersicum esculentum* (Arshad et al., 2014). Similarly, growth inhibition by increasing expression levels of *rol*B was observed in *Rubia cordifolia* callus. Other growth inhibition effects, like early leaf necrosis in *rol*B transgenic plants were previously reported in *N. tabacum* and *A. thaliana* (Schmülling et al., 1988; Nilsson et al., 1993a; Dehio and Schell, 1994; Kodahl et al., 2016), but in this study, leaf necrosis was not observed in any of the overexpressing *rol*B lines of *K. blossfeldiana*. The absence of necrosis could be attributed to the ability to suppress reactive oxygen species (ROS) observed in light induced stress in *rol*B transformed *R. cordifolia* callus (Bulgakov et al., 2012), potentially due to *rol*B regulation of NADPH oxidase isoforms (Veremeichik et al., 2016). In addition, there is evidence that the fresh and dry weight of *rolB* lines is significantly lower than that of WT in *A. thaliana* (Kodahl et al., 2016). It has been documented that the effect of *rol* genes vary in response among different species (Welander and Zhu, 2010; Grishchenko et al., 2016; Kodahl et al., 2016; Bulgakov et al., 2018). In terms of chlorophyll content, the *rol*B lines, in the current study, did not differ but had lower content compared to WT, thus not influenced by the differential copy number and expression levels observed. Moreover, Kodahl et al. (2016) suggested that the light green color of *A. thaliana* transformed with *rol*B probably was caused by the decrease of chlorophyll content. However, these results contradict the findings obtained in *rol*B under its native promoter in *Solanum lycopersicum*, where chlorophyll a/b content and non-photochemical quenching were significantly increased as well as the upregulation of five genes involved in chloroplast function (Bettini et al., 2016a). Moreover, some studies suggested that there was no difference in chlorophyll content of *S. lycopersium* transformed with *rol*B (van Altvorst et al., 1992; Arshad et al., 2014). Additionally, there has been multiple indications that *rol*B has an impact on environmental adaptive response in plants, e.g., lower accumulation of ROS under stress by ROS scavenging enzymes (Bulgakov et al., 2012), increased peroxidases activity (Veremeichik et al., 2012) and its class III production (Shkryl et al., 2013), modulation of photosynthetic activity under far-red light (Bettini et al., 2020), resistance to light stress (Bulgakov et al., 2013), as well as pathogen infection (Arshad et al., 2014).

The dwarfism and compact habit correlated with *rol*C presence have been reported in *N. tabacum* (Schmülling et al., 1988), *Chrysanthemum morifolium* (Mitiouchkina and Dolgov, 2000), *Salpignosis siniata* (Lee et al., 1996), *Petunia* (Winefield et al., 1999), and *Pelargonium* × *domenicum* (Boase et al., 2004). Here, we showed that an extreme compact habit and reduced plant growth were present *rol*C1 showing reductions of >85% for all traits. A super-dwarfed pelargonium *35S::rol*C was also found and the extreme phenotype was attributed to the insertion of a single transgene copy (Boase et al., 2004). However, despite a consistently compact habit phenotype observed in *rol*C1 and the respective gene presence supported by sequencing, it was not possible to neither determine number of transgene insertions nor obtain consistent gene expression data for *rol*C1 due to inconsistencies in detecting its presence. Taken these together, this point to a probable chimeric line that displayed the ability to survive through multiple sub-culturing rounds on kanamycin-supplemented media and showed a consistent phenotype during the flower induction experiment. The gene expression in transgenic lines driven by the *35S* promoter is known to be unstable due to various causes, e.g., silencing, post-transcriptional modifications, but mostly because the cells are a mixture of WT and transgenic cells to different proportions across tissues (Schmülling and Schell, 1993; Komarovská et al., 2010; Marenkova et al., 2012). Moreover, multiple transformation events could potentially have generated different number of insertions into the genome as well as yielding chimeric transformants. Different tissue culture techniques, e.g., second round of shoot *de novo* organogenesis, could be implemented to avoid such scenarios (Ding et al., 2020; Malabarba et al., 2020). In terms of the number of leaves, the *rol*C1 line in our study had a reduction relative to WT. On the contrary, the number of leaves of *Petunia* transformed with *rol*C was increased (Winefield et al., 1999). Compared with WT, the leaf area of the *rol*C1 line was greatly reduced. In the study of *Petunia* (Winefield et al., 1999), the leaf area of plants transformed with *rol*C was also decreased. Additionally, the *rol*C1 exhibited the lowest chlorophyll index. Conversely, the impaired chlorophyll content seemed to directly affect plant growth. The proposed effect of rolC as a β-glucosidase is known for releasing cytokinin from its N-glucosides (Estruch et al., 1991a), but other studies have shown lower amounts of cytokinin in overexpressing *rol*C transgenic tobacco (Nilsson et al., 1993b). Therefore, the *rol*C known effect in cytokinin release appears not to be directly linked to higher cytokinin levels. It remains unclear why the expected positive effects of the *rol*C presence *in planta*, e.g., reduced senescence, was not observed in the obtained transgenic line.

The role and effects of ORF13a remain elusive in the literature. The presence of ORF13a has been detected in *Nicotiana* species, first in the genome of wild *N. glauca* (Suzuki et al., 2002), and later transcriptomic studies of *N. noctiflora* reported an ORF13a transcript being expressed in this species (Long et al., 2016), suggesting this was the result of an ancient horizontal gene transfer event in an ancestor of these species. More recently, the presence of an intact copy of ORF13a in cT-DNA of *Linaria vulgaris* was observed (Vladimirov et al., 2019). However, the function of ORF13a in those studies was not investigated in detail. When Lemcke and Schmülling (1998) overexpressed ORF13a in *N. tabacum* plants, there was no observable difference between transformed plants and WT, therefore, the effect of ORF13a was not further investigated. Nevertheless, Hansen et al. (1994) first reported SPXX DNA-binding motifs at the C terminus of the ORF13a protein sequence, suggesting a potential regulatory role. In this study, we overexpressed a truncated version of ORF13a, which excluded the SPXX-DNA binding motifs from an agropine *R. rhizogenes* strain and detected an observable difference in transformed plants compared to WT. The relative number of insertions of both ΔORF13a lines were characterized as low copy and the transcript levels were ca. 2-fold higher in ΔORF13a-2 (Tables 2, 3). In a similar fashion, ΔORF13a-1 and ΔORF13a-2 showed analogous patterns of gene expression and values in biometrical parameters as the *rol*B1 and 2, in general supporting the higher copy number connection to a weaker gene expression, thus less aberrant phenotype (Komarovská Aet al., 2010). Therefore, the plant height and diameter, number of branches and leaves, leaf area, and fresh and dry weight were higher in WT followed by ΔORF13a-1 and then ΔORF13a-2 (Figures 3A–G). As mentioned before, a higher expression level led to a more compact habit and reduced plant growth in ΔORF13a. It is still uncertain if the effects observed in this study compared to previous reports are due to the omission of the SPXX-DNA binding motif, the use of *K. blossfeldiana* or to the position where the T-DNA was integrated. However, the reproducible phenotype in both ΔORF13a lines of the same species suggests that the SPXX-DNA binding motif influences the function of ORF13a. Further studies in different plants would be required to elucidate the functional mechanism of the SPXX-motif in ORF13a.

Overexpression of ORF14 was confirmed by RT-qPCR and the expression levels were relatively low, ca. 1-fold after *KlActin* and WT standardization, with an increase in ORF14-2 with approximately 1.5-fold. Moreover, ORF14 lines relative copy number was low copy range, which is contrasting to *rol*B and ΔORF13a lines where a lower copy number was associated with high transcript accumulation. The use of ORF14 in an overexpressing cassette was reported to have no changes in the morphology of transgenic plants (Lemcke and Schmülling, 1998). Interestingly, in this study, the effect of overexpressing ORF14 was a compact habit and reduced plant growth in comparison to WT. Additionally, ORF14-1 and 2 had a compactness level to the same extent as the most compact habit lines of *rol*B and ΔORF13a despite the display of increase in the respective gene expression. This fact opens up to more intriguing questions, e.g. ORF14 could potentially be as strong compactness denominator as *rol*B, as more light is shed on the less explored ORFs. It is important to highlight that the crescent number of leaves observed in ORF14-1 and ORF14-3 only resulted in statistically significant larger leaf area for ORF14-3, which indicates that ORF14-2 had more, but smaller leaves compared to ORF14-1. As mentioned previously, Lemcke and Schmülling (1998) did not observe a distinct phenotype for overexpressing neither ORF14 nor ORF13a. In general, it has been hypothesized that both ORF13 and ORF14 have a synergistic effect to the *rol* genes (Aoki and Syono, 1999b).

The number of flowers was reduced in the overexpressing lines compared to WT, which was expected since stunted vegetative growth is usually followed by a decrease in flower number (Christensen et al., 2008). The decrease in total flower numbers of approximately 50% in relation to WT for *rol*B1, ΔORF13a-1, and ORF14-3 is in accordance with data from Christensen et al. (2008) on *Kalanchoë* transformed with WT *R. rhizogenes.* However, *rol*B2, ΔORF13a-1, ORF14-1, and ORF14-2 showed approximately 85% less flowers in relation to WT, while *rol*C1 showed 99% reduction. However, in contrast, a large number of flowers were observed in chrysanthemum transformed with *rol*C (Mitiouchkina and Dolgov, 2000). Despite the lower number of flowers, a decrease in ornamental value was not observed for the *rol*B1, ΔORF13a-1, and ORF14-3 since these lines had flower numbers proportionally reduced according to the overall vegetative growth decrease. The number of flowers in tomato plants transformed with *rol*A and *rol*B was influenced by the cultivar ploidy levels, where 4x plants exhibited low flower production, i.e., 0–50 flowers, while diploid showed 100 to over 400 flowers (van Altvorst et al., 1992). In *A. thaliana*, the number of inflorescences did not differ between *rol*B and WT; however, the ratio inflorescence:leaf area revealed that *rol*B had a larger number of inflorescences per rosette area (Kodahl et al., 2016). The altered phenotype in *rol*A, *rol*B, and *rol*C transgenic *Glycine max* plants induced earlier flowering with fewer flowers than control plants (Zia et al., 2010). In natural transformation with *R. rhizogenes*, delayed flowering is an often observed significant drawback. Early or unaltered anthesis is an important factor for commercial production of ornamental plants, as delayed flowering implies in longer production span, hence higher costs. In the present study, WT had its first open flower after 68.9 ± 0.9 days and there was no alteration in flowering time for *rol*B1, *rol*B2, ΔORF13a-1, and ORF14-3. However, 12 days anthesis delay was observed for ΔORF13a-2, ORF14-1, and ORF14-2 and ultimately a 24 days postponement was observed for *rol*C1 (Figure 4C). On the contrary, other *rol*C transformed species, such as *Solanum tuberosum* (Fladung, 1990; Schmülling et al., 1993), *Atropa belladonna* (Kurioka et al., 1992), and *Osteospermum* (Giovannini et al., 1999; Allavena et al., 2000), had the characteristics of early flowering. The presence of *rol* genes/ORFs revealed substantial variation in flowering time, i.e., early, non-altered, or late flowering have been reported. The ‘Molly’ cultivar transformed with unmodified *R. rhizogenes* showed that most F1 and F2 progeny obtained by selfing displayed either later or unmodified anthesis onset, the exception was 2,202 lines that had earlier flowering than WT (Lütken et al., 2012b). The above indicated that, in regard to anthesis, the presence of the complete T-DNA can be compensated by a single *rol* gene/ORF in an overexpressing setup. The flowering period was either equal or decreased for the transformed lines compared to WT (Figure 4D). In contrast, *K. blossfeldiana* ‘Molly’ carrying the complete T-DNA, in self-crossed F1 and F2 populations, displayed a number of lines with early flowering (Lütken et al., 2012b). The *35S* promoter effect in the current study seemed to reduce the potential early anthesis effect observed by Lütken et al. (2012b). The *K. blossfeldiana* ‘Molly’ flower diameter remained unchanged despite the presence of overexpressing *rol* genes/ORFs (Figure 4E). Conversely, the same species naturally transformed with *R. rhizogenes* showed reduced flower diameter in both F1 and F2 progenies to a partial extent of the obtained crossings (Lütken et al., 2012b). Furthermore, it has been reported that pelargonium transformed with *rol*C exhibited characteristics of reduced petal area and flower diameter (Boase et al., 2004). To our knowledge, an alteration in the usual *Kalanchoë* 4-petal configuration was observed for the first time in the *rol*C1 line, although not significant due to the low number of flowers observed in this line (Figure 4F). Interestingly, it was found that the flower shape of the *rol*C line of chrysanthemum changed and the petals became wider (Mitiouchkina and Dolgov, 2000). Other studies have shown reduction in flower diameter (Lütken et al., 2012b; Kodahl et al., 2016) as well as alteration in pistil morphology, e.g., hyperstyly (Sinkar et al., 1988; van Altvorst et al., 1992).

Flower longevity and ethylene tolerance of flowers are important quality indexes of potted flowering plants. Ethylene tolerance or at least low ethylene sensitivity is necessary, especially for *K. blossfeldiana*, which is known for its sensitivity to ethylene (Woltering, 1987; Müller et al., 1998; Serek and Reid, 2000). Interestingly, in the present study, detached flowers of ORF14-3 showed increased tolerance to exogenous ethylene compared to WT and all other overexpressing lines. Additionally, other ORF14 lines, i.e., 1, 2, and 4, as well as ΔORF13a-1 showed less prominent but still improved ethylene tolerance compared to WT. Increased tolerance to ethylene was also detected in detached flowers of *K. blossfeldiana* transformed with unmodified *R. rhizogenes*, therefore carrying the full T-DNA containing all *rol* genes and ORFs (Christensen and Müller, 2009). The investigation of ethylene tolerance in Ri flowers has been previously discussed in terms of the presence of the *rol*C gene and a lower production of ethylene in these plants (Martin-Tanguy et al., 1993) as well as its cytokinin-like effect in plants (Schmülling et al., 1988; Zuker et al., 2001; Casanova et al., 2003). Moreover, there is the known alteration in source-sink relationship that led to higher glucose and sucrose levels in *rol*C and *ipt* transformed plants (Hall, 1992; Yokoyama et al., 1994; Veena and Taylor, 2007; Mohajjel-Shoja et al., 2011). Collectively, it indicated a strong connection to increased flower longevity due to a potentially higher sucrose flower content linked to a higher ethylene tolerance found in other ornamentals, e.g., cut (Kazuo et al., 2015) and potted (Monteiro et al., 2002) roses, as well as the downregulation of petal senescence associated genes (Hoeberichts et al., 2007). The present study was not able to investigate the ethylene and overexpression of *rol*C relation due to the extremely low flower production in this line (Figure 4A). Interestingly, the most relevant tolerance to ethylene was found in overexpressing ORF14 lines. This is new data as to the best of our knowledge as the ORF14 literature only describes its role as an accessory sequence that mildly mediated rooting in synergy with *rol*B (Aoki and Syono, 1999b).

The subcellular localization of a protein is physiologically important and can provide insights for future functional characterization. In this study, we conducted transient expression in *N. benthamiana* and show the subcellular localization of two proteins causing significant phenotype alterations in *K. blossfeldiana*: ΔORF13a and rolC. We present that ΔORF13a is a soluble protein that fills the cytoplasm and can permeate into the nucleus. There are according to our knowledge no studies on the subcellular localization of ORF13a to compare our results with. It is therefore unknown if the SPXX-motif repeats in ORF13a would influence localization since these motifs have DNA-binding properties, hence further investigation is needed. We observed that rolC is also a soluble protein that fills the cytoplasm able to permeate into the nucleus. Immunolocalization performed by Estruch et al. (1991b) also reported rolC as a soluble protein in the cytoplasm. Lalonde et al. (2003) reported the same, by tagging rolC with eGFP on its C-terminal end; however, they did not construct an eGFP fusion in the N-terminal end of rolC. We showed that when the position of the eGFP tag on rolC was moved from the C-terminal to the N-terminal end, rolC remained a soluble protein, but was no longer detected inside the nucleus and was found in the nuclear membrane. Proteins localized to the nuclear membrane typically display localization to the ER membrane network as well, but our experiments with co-expression of an ER/nuclear membrane control (SbCYP89A1-mRFP1) and eGFP-rolC in *N. benthamiana* leaves showed that rolC is indeed soluble and eGFP does not interfere with a localization signal to the ER membrane network. Given the size of rolC (~20KD) and of eGFP (~37KD), the fusion of these proteins could passively diffuse through the nuclear pore complex (Timney et al., 2016), therefore, it is likely that the eGFP fusion at the C-terminal end of rolC affects either a nuclear export signal or a protein-protein interaction domain of rolC with a protein in the nuclear membrane (Weill et al., 2019). These results can hopefully provide the basis for greater insights through further investigations on the functional characterization of *rol* genes and other ORFs derived proteins.

## Conclusion

We have found distinct phenotypes when individual *rol* genes and other ORFs were transformed into *Kalanchoë*, contributing to substantial changes in ethylene tolerance. The ORF14 was consistently more tolerant to this hormone and presents a new target toward ethylene tolerance studies in both Ri and overexpressing *rol* gene/ORF lines. A higher expression level combined with a single or lower number of gene copy insertions resulted in a more compact habit and reduced plant growth phenotype in *rol*B, ΔORF13a, and ORF14 lines. Additionally, novel protein data document that ΔORF13a is a soluble protein that permeates to the nucleus, while rolC-eGFP tag in the N-terminal end aborts its localization to the nuclear membrane. The increased knowledge about the differential effect of selected individual *rol* genes and previously unknown ORFs contributes to further development of *R. rhizogenes*-based transformation as a breeding tool in horticulture and agriculture. Collectively, a strong modulation of the compacted habit phenotype in terms of the distinguished effects each individual *rol* gene and selected ORFs display was found. This affected flower number and size, plant compactness as well as timing of anthesis, flower longevity, and ethylene tolerance.

## Data Availability Statement

The datasets presented in this study can be found in online repositories. The names of the repository/repositories and accession number(s) can be found in the article/Supplementary Material.

## Author Contributions

BF, AA, and HL conceptualized the present idea, planned the experiments, and contributed to the interpretation of results. BF, YT, YL, HH, NS, JX, and AA performed the experimental work and data processing. YT, YL, HH, NS, and JX documented the images presented. BF and AA wrote the original manuscript and organized the figures and tables. BF, JH, RM, AA, and HL provided critical feedback to the original manuscript editing. All authors contributed to the article and approved the submitted version.

### Conflict of Interest

The authors declare that the research was conducted in the absence of any commercial or financial relationships that could be construed as a potential conflict of interest.
