# Supplementary material for: Transgenic Kalanchoë blossfeldiana, Containing Individual rol Genes and Open Reading Frames Under 35S Promoter, Exhibit Compact Habit, Reduced Plant Growth, and Altered Ethylene Tolerance in Flowers
Source: Front Plant Sci. 2021 May 7;12:672023. doi: 10.3389/fpls.2021.672023 (PMC8138453; doi:10.3389/fpls.2021.672023)
Supplement: Supplementary file 1 [file Data_Sheet_1.docx]

Supplementary Material

## Supplementary Tables

**Supplementary table 1.** List of primers targeting full-length or fragments of *rol*B, *rol*C, ΔORF13a and ORF14, reference gene sequences and sequencing primers. *att*-overhangs are indicated in bold, USER-overhangs to clone the cassette inside pCAMBIA1300UeGFP or pCAMBIA1300UmRFP1 are indicated underlined and CC are added to maintain the reading frame.

| Target | Sequence | Amplicon size |
| --- | --- | --- |
| **Full-length** |  |  |
| *rol*B forward | 5’-GGGG**ACAAGTTTGTACAAAAAAGCAG**  **GCT**TCATGGATCCCAAATTGCTATTC-3’ | 838 bp |
| *rol*B reverse | 5’-GGGG**ACCACTTTGTACAAGAAAGCTG**  **GGT**CTTAGGCTTCTTTCTTCAGGTT-3’ |  |
| *rol*C forward | 5’-GGGG**ACAAGTTTGTACAAAAAAGCAG**  **GCT**TCATGGCTGAAGACGACCTGTGT-3’ | 601 bp |
| *rol*C reverse | 5’-GGGG**ACCACTTTGTACAAGAAAGCTG**  **GGT**CTTAGCCGATTGCAAACTTGCA-3’ |  |
| ΔORF13a forward | 5’-GGGG**ACAAGTTTGTACAAAAAAGCAG**  **GCT**TCATGCTCACCGCCGGCTGCCGA-3’ | 212 bp |
| ΔORF13a reverse | 5’-GGGG**ACCACTTTGTACAAGAAAGCTG**  **GGT**CTCAAAGCGGTTGGCTTGTAAC-3’ |  |
| ORF14 forward | 5’-GGGG**ACAAGTTTGTACAAAAAAGCAG**  **GCT**TCATGGCAGATGAGTTGGAGCGT-3’ | 616 bp |
| ORF14 reverse | 5’-GGGG**ACCACTTTGTACAAGAAAGCTG**  **GGT**CTTACATAAATACACTCTTTCC-3’ |  |
| **Fragment** |  |  |
| *rol*B forward | 5’-CCAATCTGAGCACCACTCCT-3’ | 182 bp |
| *rol*B reverse | 5’-AATCCCGTAGGTTTGTTTCG-3’ |  |
| *rol*C forward | 5’-CAATAGAGGGCTCAGGCAAG-3’ | 202 bp |
| *rol*C reverse | 5’-CCTCACCAACTCACCAGGTT-3’ |  |
| ΔORF13a forward | 5’-GGCTTGTAACGGACCTTGTG-3’ | 125 bp |
| ΔORF13a reverse | 5’-CGACGGGGAAATATGTTCTT-3’ |  |
| ORF14 forward | 5’-CGCAAAAGTAACCTCGCTTC-3’ | 245 bp |
| ORF14 reverse | 5’-CACTCTGATCCTGTGGCTGA-3’ |  |
| **Reference gene** |  |  |
| *KdActin* forward | 5'-GACTATGAGGCTGAGTTGGAGAC-3' | ~140 bp |
| *KdActin* reverse | 5'-TCAATGAAGGCTGGAAAAGG-3' |  |
| **Sequencing** |  |  |
| *pK2GW7_35S*_Fw1 | 5'-GGAAACCTCCTCGGATTCCATTG-3' | variable |
| *pK2GW7_*RB_Rv1 | 5'-AGGCGGGAAACGACAATCTGATCC-3' |  |
| *pK2GW7_35S*_Fw2 | 5'-GGAAGTTCATTTCATTTGGAGAGG-3' | variable |
| *pK2GW7_T35S*_Rv2 | 5'-AAGAACCCTAATTCCCTTATCTGG-3' |  |
| **Subcellular localization** |  |  |
| *rolC F_User* | 5'-GGCTTAAUatggctgaagacgacctgt-3' |  |
| *rolC RN_User* | 5'-GGTTTAAUttagccgattgcaaacttgcac-3 | 559 bp |
| *rolC RC_User* | 5'-GGTTTAAUCCgccgattgcaaacttgcac-3' | 561 bp |
| *ΔORF13a F_User* | 5'-GGCTTAAUatgctcaccgccggctgc-3' |  |
| *ΔORF13a RN_User* | 5'-GGTTTAAUtcaaagcggttggcttgtaacggac-3' | 170 bp |
| *ΔORF13a RC_User* | 5'-GGTTTAAUCCaagcggttggcttgtaacggac-3' | 172 bp |

**Supplementary table 2.** PCR programs used for amplification of full-length or fragment sequences of *rol*B, *rol*C, ORF13, ΔORF13a, ORF14 and reference gene sequences. All programs included an initial denaturation at 94°C for 10 min and a final elongation at 72°C for 7 min steps.

| Target(s) | PCR step | Temperature (°C) | Duration | Cycles |
| --- | --- | --- | --- | --- |
| Full-length *rol*B, *rolC* and ORF14 | Denaturing | 94 | 30 s | 35 |
|  | Annealing | 59 | 30 s |  |
|  | Elongation | 72 | 60 s |  |
| Full-length ΔORF13a | Denaturing | 94 | 30 s | 35 |
|  | Annealing | 55 | 30 s |  |
|  | Elongation | 72 | 15 s |  |
| Fragment *rol*B, *rol*C, ΔORF13a and ORF14 | Denaturing | 94 | 30 s | 35 |
|  | Annealing | 56 | 30 s |  |
|  | Elongation | 72 | 20 s |  |
| Fragment *KdActin* | Denaturing | 94 | 30 s | 35 |
|  | Annealing | 56 | 30 s |  |
|  | Elongation | 72 | 15 s |  |
| Sequencing | Denaturing | 94 | 30 s | 35 |
|  | Annealing | 55.5 | 30 s |  |
|  | Elongation | 72 | 30-80 s |  |
| Subcellular localization | Denaturing | 94 | 30 s | 35 |
|  | Annealing | 57 | 30 s |  |
|  | Elongation | 72 | 40 s |  |

**Supplementary table 3.** Media composition for transformation and tissue culture of leaf explants of *Kalanchoë blossfeldiana* 'Molly'.

| Medium | Composition† |
| --- | --- |
| Agrobacterium growth | Luria Bertani (LB) medium [10 g L^-1^ tryptone, 5 g L^-1^ yeast extract, 10 g L^-1^ NaCl, autoclaved], 75 mg L^-1^ spectinomycin and 100 mg L^-1^ rifampicin. |
| Inoculation | LB medium, 75 mg L^-1^ spectinomycin, 100 mg L^-1^ rifampicin, 15 mg L^-1^ acetosyringone |
| Washing | 1 g L^-1^ ticarcillin and clavulanic acid 15:1 ratio (timentin) |
| Co-cultivation | Murashige and Skoog medium with Gamborg vitamins (MS), 30 g L^-1^ sucrose, pH 6.3, 5 g L^-1^ gelrite, 15 mg L^-1^ acetosyringone |
| De novo organogenesis | MS medium, 30 g L^-1^ sucrose, pH 6.3, 5 g L^-1^ gelrite, 2 mg L^-1^ thidiazuron or benzylaminopurine (BAP), 200 mg L^-1^ timentin, 100 mg L^-1^ kanamycin |
| Rooting | MS medium, 30 g L^-1^ sucrose, pH 6.3, 5 g L^-1^ gelrite, 200 mg L^-1^ timentin, 100 mg L^-1^ kanamycin |

† MS medium with Gamborg vitamins Duchefa Biochemie B.V., Haarlem, The Netherlands; Antibiotics, plant growth regulators and other chemicals were from Sigma-Aldrich, St. Louis, MO, USA.

**Supplementary table 4.** Growth conditions during the experimental procedures of *Kalanchoë blossfeldiana* 'Molly' transformation, regeneration, propagation and phenotyping.

| Step | Growth condition |
| --- | --- |
| Co-cultivation | Two days in darkness at 22±3°C |
| Callus induction | 15 days in darkness at 22±3°C |
| Long day tissue culture | Climate chamber with photoperiod of 16/8h (light/dark) at 22±2°C with photosynthetic active radiation (PAR) of 250 μmol m^-2^ s^-1^ (Philips SON-T, Amsterdam, The Netherlands) until rooted shoots appearance |
| Long day climate chamber | Photoperiod 16/8h (light/dark) at 25±2°C with an approximate PAR of 500-600 μmol m^-2^ s^-1^ (Philips SON-T) for 3-4 weeks for transitioning from a closed and humidity saturated tissue culture environment, passing to a semi-covered pot to an open pot |
| Long day greenhouse | Greenhouse at 20°C set to ventilate when T ≥ 22°C, with minimum of 16h light period, natural sun light providing PAR between 700-1000 μmol m^-2^ s^-1^ supplemented with extra light if PAR<700 μmol m^-2^ s^-1^ |
| Short day greenhouse | Photoperiod 10/14 (light/dark) at 19±2°C from 7 am to 8 pm and 21±2°C from 8 pm to 7 am with an approximate PAR of 150 μmol m^-2^ s^-1^ (Philips SON-T) |
| Short day climate chamber | Photoperiod 10/14 (light/dark) at 17±2°C from 7 am to 8 pm and 21±2°C from 8 pm to 7 am with an approximate PAR of 150 μmol m^-2^ s^-1^ (Philips SON-T) |
| Ethylene climate chamber | Photoperiod 12/12 (light/dark) at 20±2°C with an approximate PAR of 20-40 μmol m^-2^ s^-1^ (Philips SON-T) |
